# Temporal and Spatial Simulation of Atmospheric Pollutant PM2.5 Changes and Risk Assessment of Population Exposure to Pollution Using Optimization Algorithms of the Back Propagation-Artificial Neural Network Model and GIS

**DOI:** 10.3390/ijerph121012171

**Published:** 2015-09-29

**Authors:** Ping Zhang, Bo Hong, Liang He, Fei Cheng, Peng Zhao, Cailiang Wei, Yunhui Liu

**Affiliations:** 1 School of Environmental and Chemical Engineering, Xi’an Polytechnic University, Xi’an 710048, China; E-Mails: miracle1891@126.com (P.Z.); mirage3000@163.com (C.W.); 2 College of Landscape Architecture and Arts, Northwest A & F University, Yangling 712100, China; 3 Xi’an Environmental Monitoring Station, Xi’an 710054, China; E-Mail: he121@163.com; 4 Forestry College, Guangxi University, Nanning 530004, China; E-Mail: ivan-025@163.com; 5 College of Life Sciences, Northwest University, Xi’an 710069, China; E-Mail: pengzhao@nwu.edu.cn; 6 College of Resources and Environmental Sciences, China Agricultural University, Beijing 100193, China; E-Mail: liuyh@cau.edu.cn

**Keywords:** PM2.5, simulation and prediction, BP-ANN model, optimization algorithms, geographical information system, population exposure risk

## Abstract

PM2.5 pollution has become of increasing public concern because of its relative importance and sensitivity to population health risks. Accurate predictions of PM2.5 pollution and population exposure risks are crucial to developing effective air pollution control strategies. We simulated and predicted the temporal and spatial changes of PM2.5 concentration and population exposure risks, by coupling optimization algorithms of the Back Propagation-Artificial Neural Network (BP-ANN) model and a geographical information system (GIS) in Xi’an, China, for 2013, 2020, and 2025. Results indicated that PM2.5 concentration was positively correlated with GDP, SO_2_, and NO_2_, while it was negatively correlated with population density, average temperature, precipitation, and wind speed. Principal component analysis of the PM2.5 concentration and its influencing factors’ variables extracted four components that accounted for 86.39% of the total variance. Correlation coefficients of the Levenberg-Marquardt (trainlm) and elastic (trainrp) algorithms were more than 0.8, the index of agreement (IA) ranged from 0.541 to 0.863 and from 0.502 to 0.803 by trainrp and trainlm algorithms, respectively; mean bias error (MBE) and Root Mean Square Error (RMSE) indicated that the predicted values were very close to the observed values, and the accuracy of trainlm algorithm was better than the trainrp. Compared to 2013, temporal and spatial variation of PM2.5 concentration and risk of population exposure to pollution decreased in 2020 and 2025. The high-risk areas of population exposure to PM2.5 were mainly distributed in the northern region, where there is downtown traffic, abundant commercial activity, and more exhaust emissions. A moderate risk zone was located in the southern region associated with some industrial pollution sources, and there were mainly low-risk areas in the western and eastern regions, which are predominantly residential and educational areas.

## 1. Introduction

The risk of population exposure to airborne particulate pollutant PM2.5 has important scientific and practical significance for sustainable development [1,2,3]. The accurate forecast of PM2.5 is critical to its prevention and control [4,5,6], and simulation and prediction of the temporal and spatial variation of PM2.5 can improve atmospheric forewarning mechanisms.

The temporal and spatial variation of PM2.5 is influenced by source and migration factors of the pollutant. Traditional methods of sentinel surveillance to obtain PM2.5 concentrations entail high equipment costs, and the coverage range of monitored sites is small and spatial density is limited. It is difficult to obtain accurate temporal and spatial distribution characteristics of PM2.5 pollution; model simulation is the most efficient way to solve the problems of spatial analysis and prediction. PM2.5 concentration prediction methods include multivariate regression methods [7,8,9], genetic algorithms [10,11], grey [12] and Markov models [13,14], and artificial neural network models (ANN) [15,16]. An artificial neural network model is a network composed of a large number of neurons; compared with other forecasting models, the back propagation (BP) artificial neural network (BP-ANN) model is widely used to predict air pollution levels because of its high accuracy and ability to accurately map complex nonlinear problems [17,18,19]. However, the shortcomings of the BP-ANN model include: (1) the simulation process of the BP-ANN model is training samples (P)- target samples (T) based, which predicts the historic PM2.5 concentrations through the relationship between training and target samples, and it does not produce significant future predictions of the PM2.5 pollution concentration; (2) PM2.5 concentration is the only data used in the BP-ANN model and does not consider sources and migration of PM2.5, resulting in lower prediction accuracies and efficiencies; and (3) the improved model only considers a single algorithm and the impact of different optimization algorithms on PM2.5 simulations is not clear. To address these shortcomings, we considered the influence of PM2.5 sources and migration to construct the BP-ANN model with application of a P-T-P′-T′ simulation method; we coupled the BP-ANN model and a geographical information system (GIS) [20,21,22] to simulate and predict the temporal and spatial variation of PM2.5 concentration after model training and validation. We attempted to produce significant future PM2.5 concentration predictions using a different algorithm-optimized BP neural network model, which attempts to produce more convincing simulation results and improve forecast accuracy and efficiency.

PM2.5 poses a serious threat to human health [23,24]. Current risk assessment of population exposure to PM2.5 is mainly dependant on the monitoring of average pollutant concentration and the estimated urban population; it does not account for the spatial and temporal distribution of pollutants and dynamic nature of population and spatial differences [25,26]. Most studies have not considered the future risk of populations exposed to PM2.5 pollution; they have only evaluated risk for historical data [27]. This study considers PM2.5 pollution inhalation rate, duration of exposure to PM2.5 pollution, the risk level of the population exposed to PM2.5 pollution, and we integrate simulated future PM2.5 concentrations into the model to provide more reasonable risk assessments under future conditions.

This study considers the role of PM2.5 sources and migration using a P-T-P′-T′ simulation method to train a BP-ANN model to simulate the forecast sample, then predict the future results. We used alternative algorithms to optimize the BP-ANN model, which realized real significant predictions of PM2.5 concentrations to improve the forecasting accuracy. We applied spatial analyses to explore the temporal and spatial distribution of PM2.5 pollution and evaluate the risk of population exposure to PM2.5 pollution. This improved comprehensive approach for simulation and prediction of PM2.5 concentration and risk assessment of population exposure to pollution provides a rational basis for the sustainable development of social, economic, and environmental policies.

## 2. Materials and Methods

### 2.1. Study Area

Xi’an is located in the middle of the Weihe river basin within the Guanzhong basin (107°40′–109°49′ E, 33°42′–34°45′ N) in the Shaanxi province of China. Xi’an is a famous historical and cultural city, and a centre for nationally important scientific research, education, and industry. With a population of 8.4678 million (in 2010), it covers an area of 10,108 km^2^, with a gross domestic product (GDP) of 488.413 billion yuan (in 2013). It has 54 rivers, and annual precipitation ranges from 522.4 mm to 719.5 mm with 1646.1–2114.9 h of sunshine annually.

### 2.2. PM2.5 Concentration Simulation and Evaluation Based on BP-ANN Model

Artificial neural network (ANN) model is a simulated organizational structure and operating mechanism based on the microscopic structure and function of the human brain. The structure is composed of a large number of hierarchically organized neurons that facilitates complex functions and can portray the nonlinear systems and uncertain systems. ANN models have the advantage of parallel computing, distributed information storage, and adaptive and strong self-learning functions; it is widely used in nonlinear control scenarios.

Database construction of the BP neural network model includes the source and the migration of PM2.5 concentration data. We rewrote the MATLAB source code of the BP-ANN model based on the mathematical matrix laboratory according to the set of normalized training samples to train the network. Continuous updating of neuron connection weights and thresholds were employed until the actual output reached the limit of allowable error; upon completion, a normalized test of PM2.5 concentration was simulated. The BP-ANN model calculation process includes the following steps [28,29,30]:

(1) According to the input vector *x*, between the input layer and the hidden layer connection weights, *ω_ij_*, and the hidden layer threshold, *α*, calculate the hidden layer output, *H*:

(1) $$H_{\text{j}}=f\left(\sum_{i=1}^{n}\omega_{ij}x_{i}-\alpha_{j}\right)\left(j=1,2,\cdots ,l\right)$$

where, *f*, the hidden incentive function, has a variety of forms of expression, but usually takes the form *f(x) = 1/(1 + ex)*.

(2) The calculation of the output layer. According to output *H of* hidden layer connection weights *ω_jk_*, and the threshold *b*, application of BP-ANN model is used to calculate the predicted output *O*; *O* is calculated as follows:

(2) $$O_{\text{k}}=\sum_{j=1}^{l}H_{j}\omega_{jk}-b_{k}\left(k=1,2,\cdots ,m\right)$$

(3) Calculation errors. According to the BP artificial neural network, to predict the output *O* and the desired output *Y*, the computing network prediction error *e* is calculated as:

(3) $$e_{\text{k}}=Y_{k}-O_{k}\left(k=1,2,\cdots ,m\right)\;)$$

(4) The updated weights. According to the prediction error value *e* of BP artificial neural network, the updated connection weights *ω_ij_* and *ω_jk_* of the network are calculated as follows:

(4) $$\omega_{ij}=\omega_{ij}+\eta H_{j}\left(1-H_{j}\right)x\left(i\right)\sum_{k=1}^{m}\omega_{jk}e_{k}\left(i=1,2,\cdots ,n;j=1,2,\cdots ,l\right)$$

(5) $$\omega_{jk}=\omega_{jk}+\eta H_{j}e_{k}\left(j=1,2,\cdots ,l;k=1,2,\cdots ,m\right)$$

(5) Update the threshold. An artificial neural network prediction error, *e*, updated the hidden layer *ω* and output layer threshold *b*; the update equation is:

(6) $$\alpha_{j}=\alpha_{j}+\eta H_{j}\left(1-H_{j}\right)\sum_{k=1}^{m}\omega_{jk}e_{k}\left(j=1,2,\cdots ,l;b_{k}=b_{k}+e_{k}\left(k=1,2,\cdots m\right)\right)$$

In this study, the correlation coefficient, *r*, is used to evaluate the simulation precision of PM2.5 concentration pollution from the BP-ANN model and the modified model parameters. The correlation coefficient is calculated as follows:

(7) $$r=\frac{\sum_{i=1}^{n}\left(S_{i}-\bar{S}\right)\times \left(O_{i}-\bar{O}\right)}{\sqrt{\sum_{i=1}^{n}{\left(S_{i}-\bar{S}\right)}^{2}\times \sum_{i=1}^{n}{\left(O_{i}-\bar{O}\right)}^{2}}}$$

where *r* is the correlation coefficient, *S_i_* is the simulation data, and *O_i_* is the monitoring data. The larger the value of *r*, the more accurate are the simulation results of PM2.5 concentration simulation compared to the observed values.

Statistical indices including the index of agreement (*IA*), the mean bias error (*MBE*), and the root mean square error (*RMSE*) were also used to evaluate the BP-ANN model. The Index of Agreement is calculated according to the equation [31]:

(8) $$IA=1-\frac{\sum_{i=1}^{n}{\left(S_{i}-O_{i}\right)}^{2}}{\sum_{i=1}^{n}{\left(\left|S_{i}-O_{ave}\right|+\left|O_{i}-O_{ave}\right|\right)}^{2}}$$

where *O_ave_* is the average value of all the observed values. The index of agreement is a dimensionless index within the range of 0–1; *IA* = 0 indicates no agreement between observed and simulated values, and *IA* = 1 indicates perfect agreement between simulated and observed values.

The Mean Bias Error is calculated as follows [32]:

(9) $$MBE=\frac{1}{n}\sum_{i=1}^{n}\left(S_{i}-O_{i}\right)$$

The *MBE* is used to describe whether a model over- or under-forecasts the observation. It has the same units as the measured variable parameter, which is predicted by the model. The ideal value of *MBE* is “zero” [33]; values >0 indicate forecasts are greater than observed values, while values <0 indicate forecasts are lesser than observed values.

The Root Mean Square Error is a commonly used measure of the differences between the values extracted by a prediction model or an estimator and the observed values. *RMSE* is calculated according the equation [34]:

(10) $$RMSE=\sqrt{\frac{\sum_{i=1}^{n}{\left(S_{i}-O_{i}\right)}^{2}}{n}}$$

The *RMSE* has the same units as the observed variable parameter, which is predicted by the model. The smaller the RMSE is, the closer the simulated values are to the observed values [35].

### 2.3. BP-ANN Model Optimization and Future Predict of PM2.5 Concentration

The back propagation artificial neural network model (BP-ANN) is the most widely used neural network model. The traditional BP-ANN model application uses the gradient descent algorithm to calculate the incremental coefficient of each layer, but it has two important problems: the slow convergence speed and the local minimum of the objective function, which greatly limits the application of the BP-ANN model. We used trainrp and trainlm algorithms to optimize the BP-ANN model; they speed up the convergence rate and improve the accuracy of the model. The trainrp algorithm removes the harmful effects of the size of the partial derivative, and it uses only the symbolic representation of the derivative to update the direction, but not the size of the derivative. The learning speed of the trainlm algorithm is fast, but the memory is very large; for a medium-sized network, trainlm is the best training algorithm.

The choice of which optimization algorithm to use greatly influences the accuracy of the BP-ANN model [36,37]. Using the trainlm and trainrp algorithms to optimize the BP-ANN model allowed us to evaluate the impact of different algorithms on the simulation results of PM2.5 concentrations. According to weather patterns, atmospheric pollution, and social and economic data from 2012 to 2014, the BP-ANN model forecasts PM2.5 concentration in 2020 and 2025. This study uses the following algorithms to improve the BP-ANN model:

(1) Trainlm: Levenberg-Marquardt optimization algorithm

This method stems from the Newton method; the basic idea is to use a locally quadratic function approximation, ψ*(z)*, and then calculate the approximate minimum point of the function. The Newton iterative method formula is:

(11) $$z(k+1)=z(k)-\sigma_{k}{\left\{H(k)\right\}}^{-1}g(k)\;(k=0,1,2,\cdots )$$

where *g(k)* = *Δ*ψ*(k)* = *Δ*ψ*(z(k))* is the performance of the function ψ*(z)* gradient vector at *z(k)*, *H(k)* = *Δ2*ψ*(k)* = *Δ2*ψ*(z(k))* is the performance of the function ψ*(z)* two ladder matrix at *z(k)*, the Hesse matrix.

In general, the optimal performance function ψ*(z)* at z point in the Hesse matrix is:

(12) $$H(z)=\Delta^{2}\psi (z)=\left[\begin{array}{cccc} \frac{\partial^{2}\psi (z)}{\partial z_{1}^{2}} & \frac{\partial^{2}\psi (z)}{\partial z_{1}z_{2}} & \cdots & \frac{\partial^{2}\psi (z)}{\partial z_{1}z_{Nz}}\\ \frac{\partial^{2}\psi (z)}{\partial z_{2}z_{1}} & \frac{\partial^{2}\psi (z)}{\partial z_{1}^{2}} & \cdots & \frac{\partial^{2}\psi (z)}{\partial z_{2}z_{Nz}}\\ \cdots & \cdots & \cdots & \cdots \\ \frac{\partial^{2}\psi (z)}{\partial z_{Nz}z_{2}} & \frac{\partial^{2}\psi (z)}{\partial z_{Nz}z_{2}} & \cdots & \frac{\partial^{2}\psi (z)}{\partial z_{Nz}^{2}} \end{array}\right]$$

where, *N_z_* is the number of *z*-dimensional parameter vector.

(13) $$z(k+1)=z(k)+\sigma_{k}d\left(k\right)$$

(14) $$d(k)\;=-{\left\{\overset{\wedge }{H}\left(k\right)\right\}}^{-1}g\left(k\right)$$

(15) $$\overset{\wedge }{H}\left(k\right)\;=H\left(k\right)+\beta_{k}\overset{\wedge }{Q}\left(k\right)\;\left(\beta \geq 0;k=1,2,\cdots \right)$$

(2) Trainrp: flexible algorithm

This method eliminates the harmful effects of the size of the partial derivative of the weights by using only the direction, positive or negative, of the updated derivatives, regardless of the size of the derivatives.

(16) dX=deltaX.*sign(gX)

where, *gX* is the gradient, and *deltaX* is the weight values; repeated updates will be amended in accordance with *gX* and direction of the derivatives’ similarities and differences.

### 2.4. Risk Assessment for Population Exposure to PM2.5

We used the risk assessment method of the United States Environmental Protection Agency (EPA) [38], combined with conditions specific to China to amend some parameters, to evaluate the risk of population exposure to PM2.5 in Xi’an. The main parameters and values of population exposure risk assessment used were as follows: *C* is the PM2.5 concentration data (μg/m^3^) according to the Xi’an environmental monitoring station; the inhalation rate, *IR*, was 11.7 m^3^/day, which accounts for the population average for men, women, children, and adolescents; the unit of risk, *UR*, was 0.008 μg/m^3^; and the average body weight, *BW*, was 57 kg, which was the population average of men, women, children, and adolescents. The exposure duration (*ED*, days) and the number of days over which the exposure is averaged (*AT*, days) for non-carcinogenic effects are equal (*ED* = *AT*); thus, they cancel each other out and were not included in the model [27]. Prediction of future population dynamics used the demographic data between 2010 and 2014.

The risk characterization and risk of population exposure to PM2.5 was evaluated by the following formula [27]:

(17) $$RI_{i}=Dose\times Toxicity$$

where, *RI_i_* is pollution health risks of exposure for the individual, *Dose* the amount of potential inhalation dose, *i.e.*, the amount of material that can be used in the metabolic processes of the interaction, and *Toxicity* is the amount of toxicity.

*ADD_opt_* is the average daily dose, calculated as:

(18) $$ADD_{opt}=\left(C\times IR\times ED\right)/\left(BW\times AT\right)$$

where *C* was pollutant concentration (μg/m^3^), *IR* was inhalation rate (m^3^/day), *ED* was exposure duration (days), *BW* was weight (kg), and *T* was the average exposure days (days).

*SFI* is the inhalation of slope factor, calculated as:

(19) SFI=UR×BW×IR

where *IR* is the inhalation rate (m^3^/day), *UR* is the unit of risk (μg/m^3^), and *BW* is the body weight (kg). From *ADD_opt_* and *SFI*, we calculated *R_i_* as individual health risk for exposure pollution:

(20) $$R_{\text{i}}=ADD_{opt}\times SFI$$

*POP_risk_* is the health risks for the population exposed to pollution, calculated as:

(21) $$POP_{risk}=R_{i}\times POP_{\exp osed}$$

where *POP_exposed_* is the pollution exposure for population density.

### 2.5. Principal Component Analysis

Principal component analysis (PCA) is a multivariate statistical analysis using linear transformations of multiple variables to select fewer numbers of important variables. PCA of the input data included social data (e.g., population density), economic data (e.g., GDP), environmental data, including SO_2_, NO_2_, and PM2.5 concentrations of atmospheric pollutants, and meteorological data that included average temperature, precipitation and wind speed. The variables were of different orders of magnitude, thus each variable was normalized to unit variance. Factor analysis was conducted after checking the suitability of the dataset, and PCA with varimax rotation was used; only eigenvalues of components with greater than one unit after rotation were retained. PCA was conducted using SPSS, Version 19 for Windows XP [39,40].

### 2.6. Model Data

Air pollution data included daily PM2.5, SO_2_, and NO_2_ concentration monitoring data in 2012 and 2014 from 12 monitoring stations. Meteorological data included wind speed, air temperature, precipitation, and social (e.g., population density) and economic data (e.g., GDP).

## 3. Results and Discussion

### 3.1. Influencing Factors and PM2.5 Concentration Linkages

Correlation analyses between the influencing factors and PM2.5 concentration are shown in Table 1. GDP was significantly correlated with population density with a correlation coefficient of 0.659. Population density had a positive correlation with wind speed (*r* = 0.230), however, it was only weakly negatively correlated to PM2.5 (*r* = −0.006). Average temperature had a positive correlation with wind speed (*r* = 0.601), while it had a strong negative correlation with concentration of SO_2_ and PM2.5, with correlation coefficients of −0.831 and −0.831, respectively. Precipitation was positively correlated with average temperature (*r* = 0.494), however, it was negatively correlated to SO_2_, NO_2_, and PM2.5, with correlation coefficients of −0.485, −0.287, and −0.504, respectively (Table 1).

**Table 1:** Pearson correlation coefficients of influencing factors and PM2.5 concentration in Xi’an, China.

	GDP	Population Density	Average Temperature	Precipitation	Wind Speed	SO_2_	NO_2_	PM2.5
GDP	1.000	0.659 **	0.017	0.052	0.438 **	0.061	0.211*	0.029
Population density	0.659 **	1.000	0.009	0.027	0.230 **	0.002	0.146	−0.006
Average temperature	0.017	0.009	1.000	0.494 **	0.601 **	−0.831 **	−0.489 **	−0.831 **
Precipitation	0.052	0.027	0.494 **	1.000	0.310 **	−0.485 **	−0.287 **	−0.504 **
Wind speed	0.438 **	0.230 **	0.601 **	0.310 **	1.000	−0.469 **	−0.122	−0.454 **
SO_2_	0.061	0.002	−0.831 **	−0.485 **	−0.469 **	1.000	0.461 **	0.796 **
NO_2_	0.211 *	0.146	−0.489 **	−0.287 **	−0.122	0.461 **	1.000	0.391 **
PM2.5	0.029	−0.006	−0.831 **	−0.504 **	−0.454 **	0.796 **	0.391 **	1.000

** Significance at the 0.01 probability level. * Significance at the 0.05 probability level.

Wind speed was positively correlated with GDP and precipitation, with correlation coefficients of 0.438 and 0.310, respectively, while it was negatively correlated with SO_2_, NO_2_, and PM2.5, with correlation coefficients of −0.469, −0.122, and −0.454, respectively. SO_2_ had a positive correlation with NO_2_ and PM2.5, with correlation coefficients of 0.461 and 0.796, respectively, however, it had a strong negative correlation with average temperature (*r* = −0.831). NO_2_ had a positive correlation with SO_2_ and PM2.5, with correlation coefficients of 0.461 and 0.391, respectively, while it was negatively correlated with average temperature (*r* = −0.489). PM2.5 had a positive correlation with GDP, SO_2_, and NO_2_, with correlation coefficients of 0.029, 0.796, and 0.391, respectively, however, it was negatively correlated with population density, average temperature, precipitation, and wind speed, with correlation coefficients of −0.006, −0.831, −0.504, and −0.454, respectively. The correlation analysis showed that economic development should be accompanied by measures to protect the atmosphere, such as controlling fuel burning and automobile emissions. In addition, precipitation and wind speed appeared to be beneficial to PM2.5 migration because it maintains it at low concentrations.

PM2.5 concentration was positively correlated with GDP, SO_2_, and NO_2_, which indicates that regions with high GDP values, such as metropolitan areas, have a large proportion of urbanization and industries that produce more contaminants [25]. GDP growth would accelerate the deterioration of the environment; motor vehicle exhaust, coal dust, fuels, and secondary particles, particularly primary pollutants SO_2_ and NO_2_, may also be contributing factors to PM2.5 concentration [26,41].

PM2.5 had a negative relation with meteorological factors, including average temperature, precipitation, and wind speed. Temperature decreases with increasing altitude, and the upward movement of the lower, warmer atmosphere that contains high levels of air pollutants and dust movement spreads the contaminants to higher altitudes, thus reducing the extent of pollution in the lower atmosphere [42]. Precipitation can effectively reduce PM2.5 concentration by atmospheric wet deposition; it effectively removes particulate matter, especially smaller sized particles, from the atmosphere, and rainfall has an important role in scouring air pollutants and air purification [43]. The negative correlation between PM2.5 and wind speed is because wind speed can blow away the pollutants within a certain geographical range, but it is unable transport large quantities of pollutants from far away, which is consistent with previous findings [44]. In addition, wind speed will contribute to the dilution and diffusion of particles near the surface layer.

### 3.2. Principal Component Analysis of PM2.5 Concentration and Its Impact Factors

Principal component analysis (PCA) of the PM2.5 concentration and influencing factors variables extracted four components that accounted for 86.39% of the total variance (Table 2). The first principal component was mainly comprised of average temperature, SO_2_, PM2.5, precipitation, and wind speed, with coefficient loads of 0.94, 0.90, 0.89, 0.65, and 0.65, respectively. The second principal component was largely influenced by GDP and population density, with coefficient loads of 0.92 and 0.83, respectively. The third principal component was mainly represented NO_2_ with a coefficient load of 0.65. The fourth principal component was mainly represented precipitation with a coefficient load of 0.74. These four principal components essentially reflected the information of all indicators. It demonstrates that PM2.5 is affected by a complex suite of factors, especially the meteorological, economic, and the primary and secondary particulate matter, which had a higher contribution to PM2.5 concentration [39,40,45,46].

**Table 2:** Principal component analysis (PCA) for PM2.5 concentration and influencing factors in Xi’an, China.

	Component Matrix			
	1	2	3	4
GDP	0.06	0.92	−0.08	−0.01
Population density	0.05	0.83	−0.37	0.02
Average temperature	0.94	−0.03	0.08	−0.14
Precipitation	0.65	0.00	−0.07	0.74
Wind speed	0.65	0.48	0.32	−0.22
SO_2_	−0.90	0.11	−0.05	0.08
NO_2_	−0.56	0.38	0.65	0.22
PM2.5	−0.89	0.08	−0.11	0.01
Cumulative %	45.35	69.39	78.06	86.39

### 3.3. Validation of BP-ANN Model Based on Optimization Algorithm

**Figure 1:**
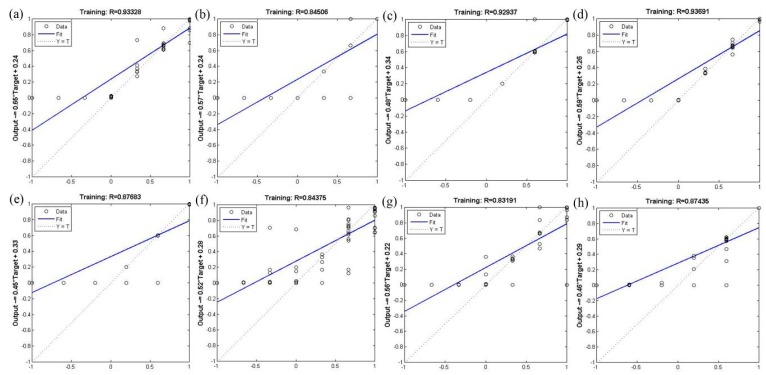

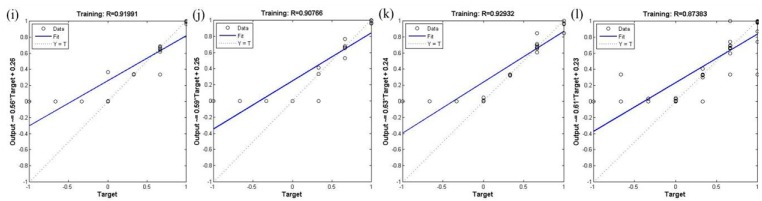
Validation of the BP-ANN model using the trainlm optimization algorithm. The 12 monitoring stations are as follows: (**a**) high-voltage switchgear plant; (**b**) xingqing district; (**c**) the textile city; (**d**) hamlet; (**e**) people’s stadium; (**f**) new district; (**g**) economic development zone; (**h**) chang’an district; (**i**) lintong district; (**j**) qujiang district; (**k**) guangyuntan; (**l**) marsh.

**Figure 2:**
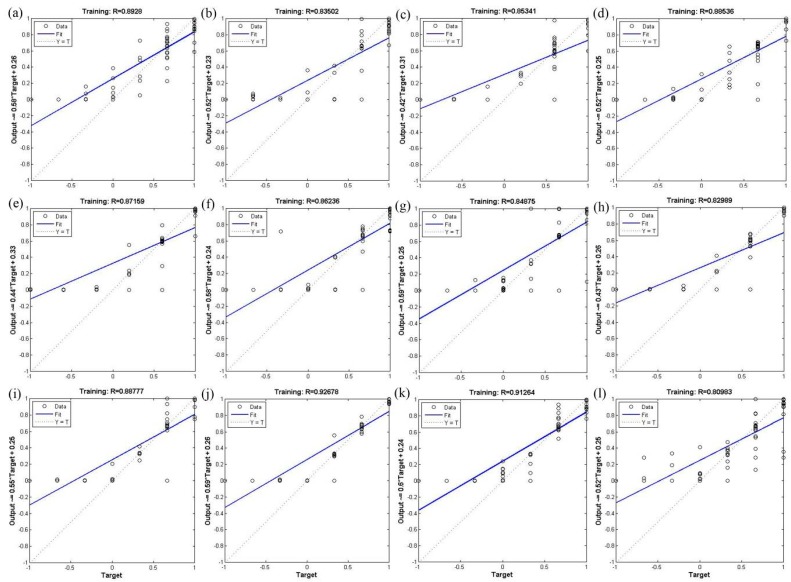
Validation of the BP-ANN model using the trainrp optimization algorithm. The 12 monitoring stations are as follows: (**a**) high-voltage switchgear plant; (**b**) xingqing district; (**c**) the textile city; (**d**) hamlet; (**e**) people’s stadium; (**f**) new district; (**g**) economic development zone; (**h**) chang’an district; (**i**) lintong district; (**j**) qujiang district; (**k**) guangyuntan; (**l**) marsh.

The correlation coefficient was used to validate the BP-ANN model; Figure 1 and Figure 2 show that the correlation coefficient of the BP-ANN model by two optimization algorithms was more than 0.8. The mean correlation coefficient of trainlm optimization algorithm in the 12 monitoring sites was 0.89; the maximum correlation coefficient was 0.94, while the minimum was 0.83 (Figure 1). The mean correlation coefficient of trainrp optimization algorithm in the 12 monitoring sites was 0.87; the maximum correlation coefficient was 0.93, while the minimum was 0.81 (Figure 2). The high correlation coefficient values indicates that the optimization algorithms used within the BP-ANN model were able to meet the model requirements and can be used for predicting the PM2.5 concentration in the study area.

The index of agreement (IA) ranged from 0.541 to 0.863 using the trainlm algorithm, and from 0.502 to 0.803 with the trainrp algorithm in the BP-ANN model, which indicates that the predicted values were very close to the observed values. Based on the mean bias error (MBE) values, the model slightly overestimates the PM2.5 concentration for all monitoring stations with the exception of one station (marsh) where the model underestimated the values. The Root Mean Square Error (RMSE) indicated only small differences between forecasted and observed PM2.5 concentration values (Table 3).

**Table 3:** Evaluation of the forecasting accuracy for PM2.5 concentration.

Station	IA		MBE		RMSE	
	Trainlm	Trainrp	Trainlm	Trainrp	Trainlm	Trainrp
High-oltage Switchgear Plant	0.748	0.717	1.3333	1.4189	1.414	1.529
Xingqing District	0.783	0.803	1.0672	1.0087	1.071	1.009
The Textile City	0.710	0.597	0.6667	1.0000	0.816	1.100
Hamlet	0.759	0.708	0.3405	0.3524	0.925	0.983
People’s Stadium	0.797	0.733	0.5344	0.6277	0.537	0.770
New District	0.556	0.559	1.7336	1.7357	1.786	1.775
Economic Development Zone	0.512	0.569	1.9280	1.6718	1.950	1.738
Chang’an District	0.580	0.695	1.3650	1.0003	1.390	1.081
Lintong District	0.767	0.502	0.3333	0.7057	0.913	1.534
Qujiang District	0.661	0.658	1.2691	1.2852	1.386	1.407
Guangyuntan	0.541	0.533	1.2085	0.9277	1.490	1.263
Marsh	0.863	0.667	−0.2352	0.0003	0.602	0.816

### 3.4. Analysis of Temporal and Spatial Simulation of Atmospheric Pollutants PM2.5 Concentration

**Figure 3:**
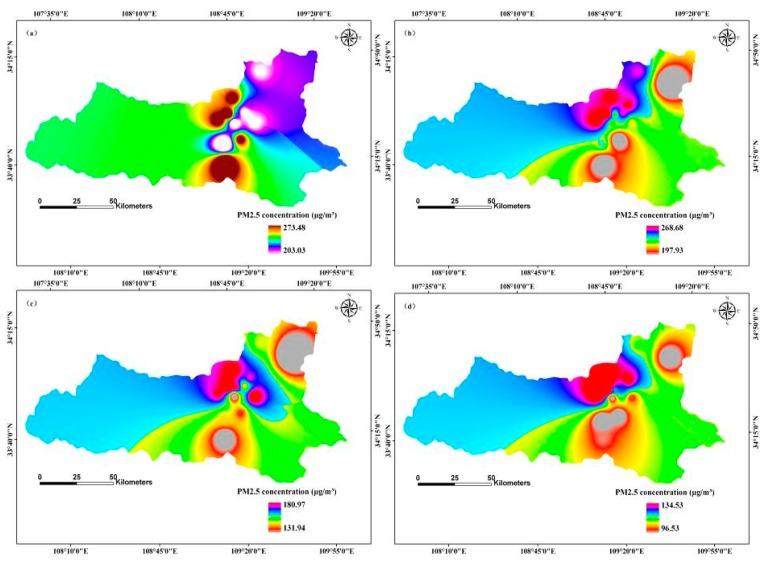

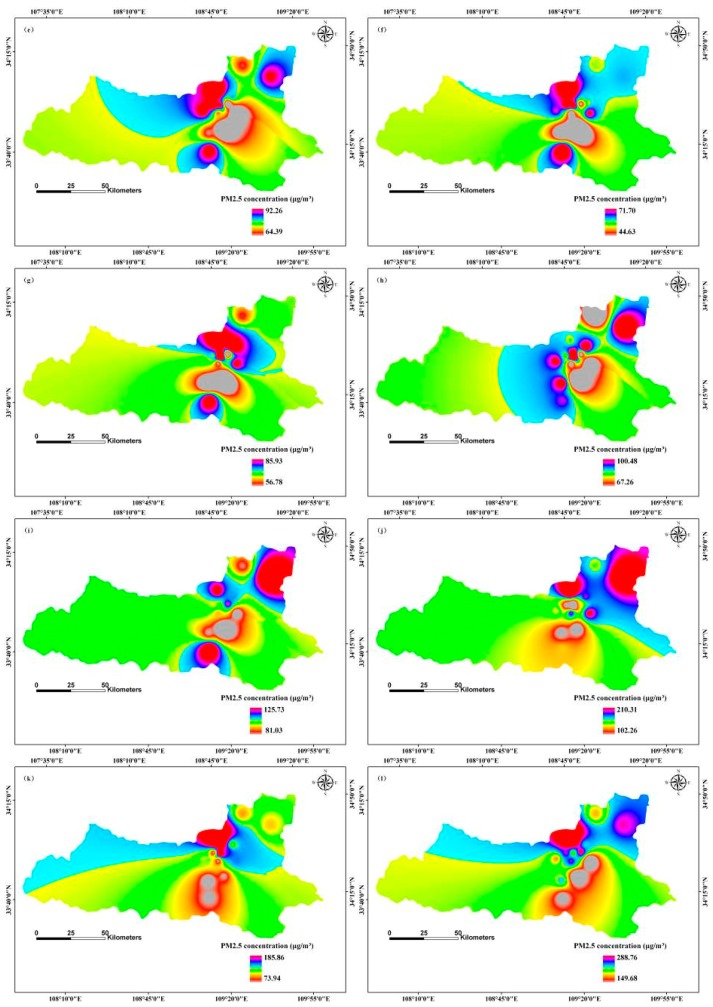
Simulation of temporal and spatial changes of PM2.5 concentration based on a BP-ANN model in 2013, Xi’an, China by month: (**a**) January; (**b**) February; (**c**) March; (**d**) April; (**e**) May; (**f**) June; (**g**) July; (**h**) August; (**i**) September; (**j**) October; (**k**) November; (**l**) December.

**Figure 4:**
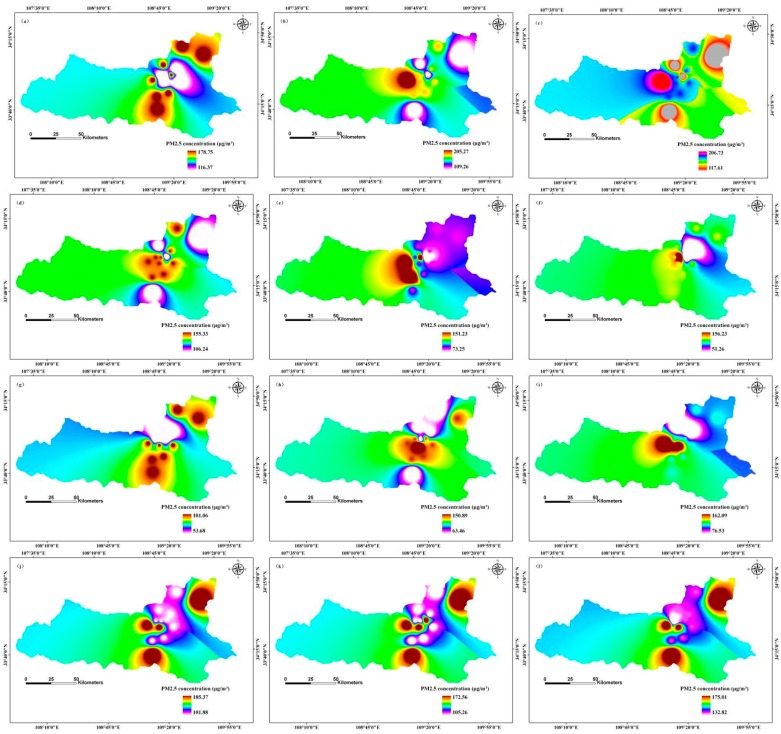
Simulation of temporal and spatial changes of PM2.5 concentration based on a BP-ANN model in 2020, Xi’an, China by month: (**a**) January; (**b**) February; (**c**) March; (**d**) April; (**e**) May; (**f**) June; (**g**) July; (**h**) August; (**i**) September; (**j**) October; (**k**) November; (**l**) December.

**Figure 5:**
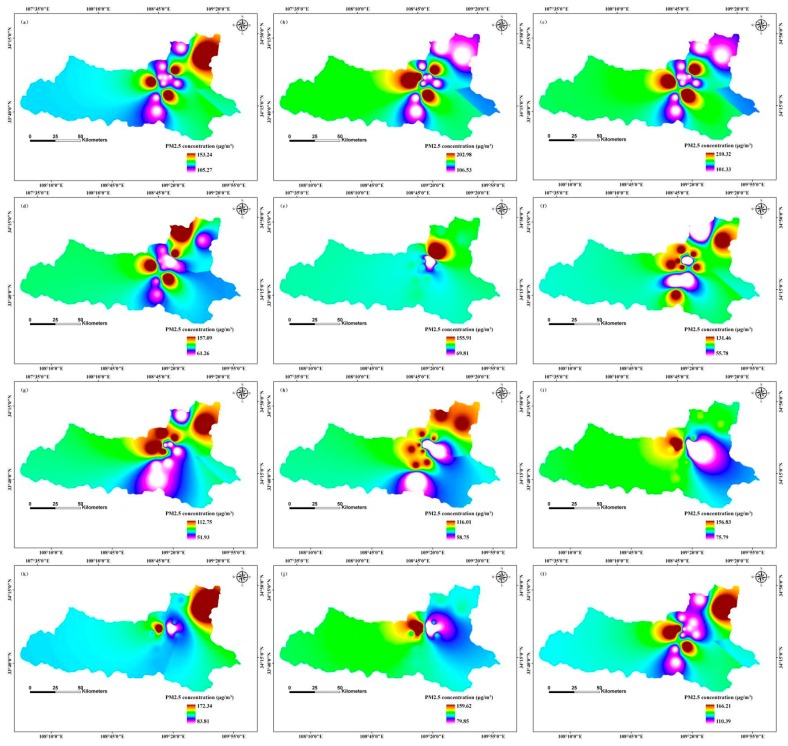
Simulation of temporal and spatial changes of PM2.5 concentration based on a BP-ANN model in 2025, Xi’an, China by month: (**a**) January; (**b**) February; (**c**) March; (**d**) April; (**e**) May; (**f**) June; (**g**) July; (**h**) August; (**i**) September; (**j**) October; (**k**) November; (**l**) December.

We applied the inverse distance weighted (IDW) interpolation method to model temporal and spatial distributions of PM2.5 concentration. Spatial distribution of monthly average simulation values clearly indicated different PM2.5 concentration distributions in different months in 2013, 2020, and 2025 (Figure 3, Figure 4 and Figure 5).

The concentration ranges of PM2.5 was between 203.03 μg/m^3^ and 273.48 μg/m^3^ in January 2013, with the maximum distribution in the north-central region, and the minimum distribution was in the southern and eastern regions; the median concentration was in the eastern region (Figure 3). In contrast, in May 2013, the range of PM2.5 concentrations was from 64.39 μg/m^3^ to 92.26 μg/m^3^, with the maximum distribution in north-central, south-central, and north-eastern regions, and the minimum distribution in the central region; the intermediate value distribution was in the south-eastern region. However, PM2.5 concentration ranged from 67.26 μg/m^3^ to 100.48 μg/m^3^ in August 2013, with the maximum distribution in the north-central and north-eastern regions, and the minimum distribution in the central and north-central regions; the median PM2.5 concentration was in the western and south-eastern regions. The PM2.5 concentration ranged from 73.94 μg/m^3^ to 185.86 μg/m^3^ in November, with the maximum distribution in the north-central region, and the minimum located in the south-central region; intermediate value distribution was in the south-western, north-eastern, and south-eastern regions (Figure 3).

Predicted values of PM2.5 concentration for February 2020 ranged from 109.26 μg/m^3^ to 205.27 μg/m^3^, with maximum values mainly distributed in the north-central region, and minimum values mainly in central and southern regions; the median PM2.5 concentration was in the western region (Figure 4). In contrast, PM2.5 concentration range in June of 2020 was 51.26 μg/m^3^ to 156.23 μg/m^3^, with the maximum distribution in the north-central region, and the minimum distribution in the north-eastern region; median values were mainly in the south-eastern region. However, PM2.5 concentration ranged from 162.09 μg/m^3^ to 176.53 μg/m^3^ in September 2020, with the maximum distribution in the north-central region, and minimum PM2.5 concentration located in the north-central region; intermediate values were in the north-eastern and south-eastern regions. While PM2.5 concentrations ranged from 132.82 μg/m^3^ to 175.01 μg/m^3^ in December 2020, the maximum distribution was in the central, south-central, and north-eastern regions, and the minimum distribution was in the north-central region; median values occurred mainly in the western region (Figure 4).

In March 2025, PM2.5 concentration ranged from 103.33 μg/m^3^ to 210.32 μg/m^3^, with maximum concentration mainly distributed in the central region, and the minimum values mainly in the north-eastern region; median PM2.5 concentration was in the western and south-eastern regions (Figure 5). In contrast, PM2.5 concentration ranged from 61.26 μg/m^3^ to 157.09 μg/m^3^ in April 2025, with the maximum distribution in the central and north-eastern regions, and the minimum distribution in north-central region; median values occurred mainly in the western region. However, PM2.5 concentration range in July 2025 was between 51.93 μg/m^3^ and 112.75 μg/m^3^, with the maximum distribution in north-central and north-eastern regions, and the minimum distribution in central and southern regions; the median values were in the western and south-eastern regions. PM2.5 concentration ranged between 79.85 μg/m^3^ and 159.62 μg/m^3^ in October 2025, with the maximum distribution in north-central region, and the minimum values were in the eastern region; intermediate values occurred mainly in the north-eastern and south-eastern regions (Figure 5).

The seasonal variation of PM2.5 concentration was as follows: winter > spring > autumn > summer. These results are consistent with the findings of Huang *et al.* [47]. Winter PM2.5 concentration is strongly influenced by coal heating, which increases particulate concentrations, and temperature inversions, which does not encourage the dispersion of particulate matter [48]. Spring concentrations are related to frequent dust storms and construction dust, both of which greatly increase particulate matter concentrations. In summer, rainy weather and windy days can increase the sedimentation ability of the air and help diffuse particulate matter. In autumn, low temperatures and low wind speeds do not contribute to the diffusion of PM2.5.

Temporal and spatial variation of PM2.5 concentration displayed a declining trend between 2013 and 2025. Government efforts to regulate PM2.5 pollution, including optimizing energy structure, promotion of green travel, and the reduction of industrial emissions, construction dust, fuel combustion, and vehicle exhaust pollution, are designed to protect public health and ensure sustainable development of the environment and economy, although they likely contributed to a reduced GDP growth rate also. Reduction of the primary pollutants SO_2_ and NO_2_ pollution also decreased the PM2.5 concentration. Decreased PM2.5 concentration was also influenced by the changes of meteorological factors [44,49,50].

The highest PM2.5 concentration distribution in the central and northern regions was largely because of downtown traffic and extensive commercial activity. The moderate risk zone located in the southern region had a small number of industrial pollution sources. The eastern and western regions were mainly residential and education areas and were far away from downtown, thus they had less pollution. In addition, the geographical locations and meteorological factors influenced PM2.5 concentrations [47].

### 3.5. Risk Assessment of Population Exposure to PM2.5 Pollution

We used a risk assessment model of population exposure to PM2.5 pollution to evaluate the risk of population exposure to PM2.5. The model considers the pollution inhalation rate and the duration of exposure to pollution; we used the natural break method to divide the area by risk level of population exposure to PM2.5 pollution. In general, the higher concentration of PM2.5 and the greater population density indicated a higher risk of population exposure.

**Figure 6:**
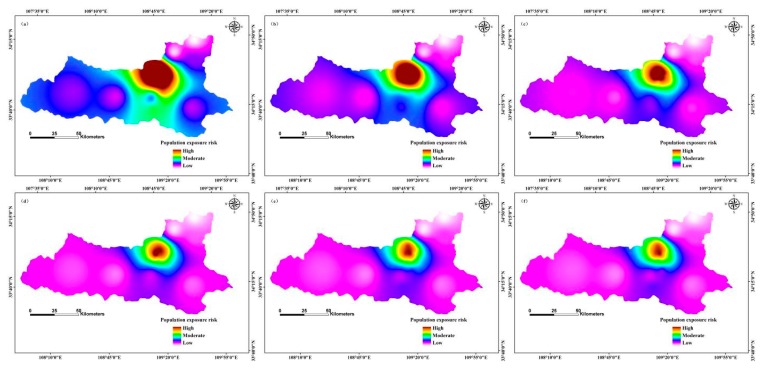

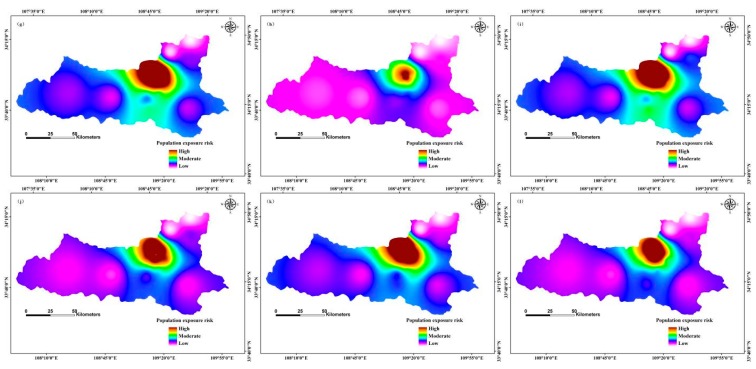
Risk of population exposure to PM2.5 in 2013 in Xi’an, China by month: (**a**) January; (**b**) February; (**c**) March; (**d**) April; (**e**) May; (**f**) June; (**g**) July; (**h**) August; (**i**) September; (**j**) October; (**k**) November; (**l**) December.

The high-risk areas of population exposure to PM2.5 were distributed in the northern regions in January 2013, and low-risk areas were in the western and north-eastern regions; moderate risk zones occurred in the southern region (Figure 6). In contrast, in March 2013, high-risk areas of population exposure to PM2.5 were distributed in the northern regions, and low-risk areas were in the western and southern regions; the moderate risk zone was located in the north-central region. In July 2013, the high-risk regions of population exposure to PM2.5 were located in the north-central region, and low-risk zones were in the north-eastern region; moderate risk areas were distributed in the central and southern regions. In August 2013, the high-risk regions of population exposure to PM2.5 were distributed in the northern regions, and low-risk areas in the north-eastern region; the moderate risk zone was located in the north-central region (Figure 6).

**Figure 7:**
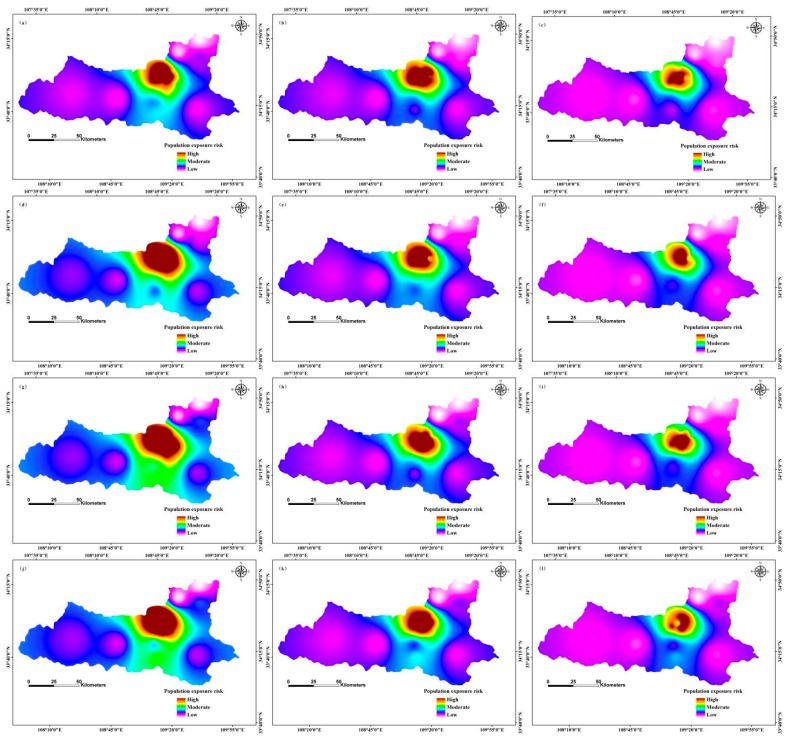
Risk of population exposure to PM2.5 in 2020 in Xi’an, China by month: (**a**) January; (**b**) February; (**c**) March; (**d**) April; (**e**) May; (**f**) June; (**g**) July; (**h**) August; (**i**) September; (**j**) October; (**k**) November; (**l**) December.

In April 2020, the high-risk areas of population exposure to PM2.5 were distributed in the north-central region, and the low-risk areas occurred in the western and north-eastern regions; moderate risk zones were located in the central and southern regions (Figure 7). In contrast, high-risk areas of population exposure to PM2.5 were distributed in the northern region in September 2020, and low-risk areas in the western and eastern regions; the moderate risk zone was located in the north-central region. In October 2020, the high-risk regions of population exposure to PM2.5 were located in the north-central region, and the low-risk zone was in the north-eastern region; moderate risk areas were distributed in the southern region. In December 2020, the high-risk regions of population exposure to PM2.5 were distributed in the northern region, and the low-risk areas were in the western and eastern regions; the moderate risk zone was located in the north-central region (Figure 7).

**Figure 8:**
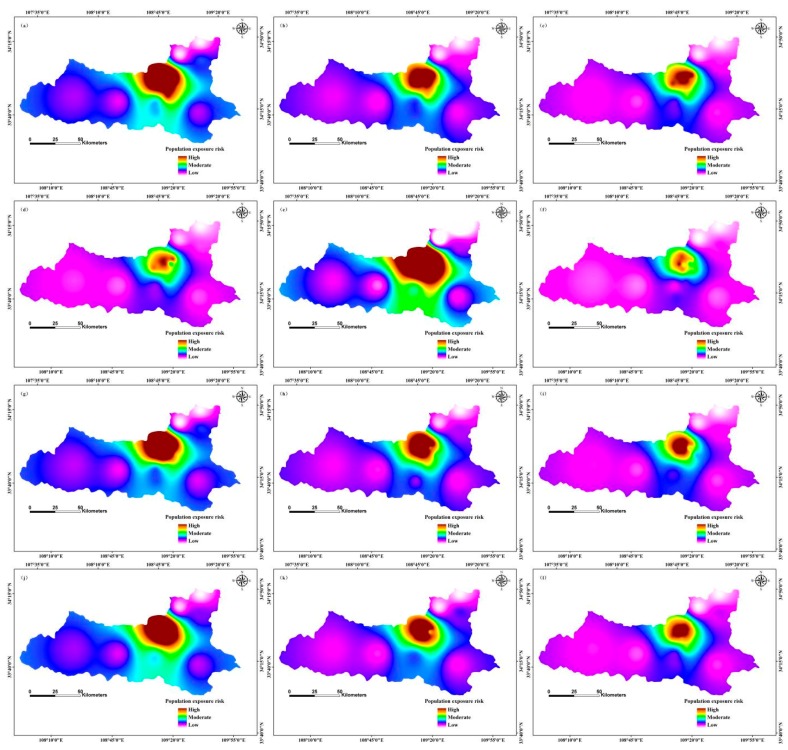
Risk of population exposure to PM2.5 in 2025 in Xi’an, China by month: (**a**) January; (**b**) February; (**c**) March; (**d**) April; (**e**) May; (**f**) June; (**g**) July; (**h**) August; (**i**) September; (**j**) October; (**k**) November; (**l**) December.

In May 2025, the high-risk areas of population exposure to PM2.5 were distributed in the north-central region, and the low-risk areas occurred in the western and north-eastern regions; the moderate risk zones were in the south-central region (Figure 8). In June 2025, high-risk areas of population exposure to PM2.5 were distributed in the northern region, and low-risk areas were located in the western and eastern regions; moderate risk zones were located in the north-central region. In July 2025, the high-risk regions of population exposure to PM2.5 were located in the north-central region, and the low-risk zones occurred in the western and north-eastern regions; the moderate risk areas were distributed in the southern region,. In November 2025, the high-risk regions of population exposure to PM2.5 were distributed in the northern region, and low-risk areas were in the western and north-eastern regions; the moderate risk zones were located in the north-central region (Figure 8).

Areas of high PM2.5 concentration had a high population exposure risk, and areas of high population density had correspondingly higher exposure risk. Our results indicate that the spatial distribution of PM2.5 concentration is consistent with the risk of population exposure risk [26,51]. Other scholars have used different methods to study the risk of population exposure to PM2.5. For example, Yao *et al.* [25] used a population exposure risk model by taking into account the population density in mainland China; their results are consistent with our findings. Researchers have also used the area-weighting method to estimate population exposure to PM2.5 in Beijing, China; that study revealed relationships between long-term overexposure to PM2.5 and people living in high exposure areas of Beijing [26]. The researchers applied the population exposure risk model to different study areas, which provided a scientific basis for the treatment of air pollution. However, in the future, we propose that researchers should further compare and analyse the simulation results of different models, and adopt different methods to optimize the model.

In this study, the optimized time series of the BP artificial neural network model combined with GIS spatial analysis technology allowed for the simulation and prediction of temporal and spatial distribution of PM2.5 concentrations and population exposure risk five and 10 years into the future. Unlike studies that examine historical patterns of PM2.5 concentration simulation [47], this study models future predictions. Compared with the short-term forecasts [52], our forecast period is longer and the precision is higher.

However, this study also has some shortcomings. Firstly, the BP artificial neural network model is a time series model that does not account for the spatial distribution of land use and population. The simulation and forecast of PM2.5 concentration in subsequent studies should be combined with land use and population distribution data. Secondly, this study uses the inverse distance weighted interpolation method to model the spatial distribution of PM2.5 concentration based on limited data (12 monitoring stations) to predict the value of other units. This approach requires that the discrete uniform distribution and sufficient point density reflect the local surface in the analysis. However, if the distance or the power value is bigger, it may generate incorrect results. Compared with the PM2.5 simulation method by Aerosol Optical Depth (AOD), the precision of IDW is lower; therefore, we should further strengthen the spatial aspects of PM2.5 and AOD research to improve modelling and risk assessments.

## 4. Conclusions

In this study, the optimized BP-ANN model and geographical information system were used to simulate and predict the temporal and spatial changes of atmospheric pollutant PM2.5 and analyse the risk of population exposure to PM2.5. Our main conclusions are:

(1) Correlation analyses indicated that PM2.5 had a positive correlation with GDP, SO_2_, and NO_2_, however, it was negatively correlated to population density, average temperature, precipitation, and wind speed.

(2) Principal component analysis indicated that four principal components accounted for 86.39% of the total variance; the first principal component (45.4% of total variance) mainly represented average temperature, SO2, PM2.5, precipitation, and wind speed.

(3) The correlation coefficient of the BP-ANN model by two optimization algorithms was more than 0.8, which demonstrates that the BP-ANN model can be used to predict PM2.5 concentrations in the study area; accuracy of the trainlm algorithm was higher than the trainrp algorithm.

(4) Compared with 2013, PM2.5 concentration and risk of population exposure to pollution were decreasing in 2020 and 2025. PM2.5 concentration ranged from 67.26 μg/m^3^ to 100.48 μg/m^3^ in August 2013, with the maximum distribution in north-central and north-eastern regions, and the minimum distribution in the central and north-central regions; the median PM2.5 concentration was in the west and south-eastern regions. However, June 2013 PM2.5 concentrations ranged from 51.26 μg/m^3^ to 156.23 μg/m^3^, with the maximum distribution in the north-central region, and the minimum distribution in the north-eastern region; the median PM2.5 concentrations occurred mainly in the south-eastern region. In contrast, PM2.5 concentration ranged from 79.85 μg/m^3^ to 159.62 μg/m^3^ in October 2013, with the maximum distribution in the north-central region, and the minimum distribution in the eastern region; intermediate values were mainly in the north-eastern and south-eastern regions. In January 2013, the high-risk areas of population exposure to PM2.5 were distributed in the northern region, and low-risk areas were in the western and north-eastern regions; the moderate risk zone was in the southern region. In contrast, in April 2020, the high-risk areas of population exposure to PM2.5 were distributed in the north-central region, and low-risk areas were in the western and north-eastern regions; the moderate risk zone was in the central and southern. In November 2025, the high-risk regions of population exposure to PM2.5 were distributed in the northern region, with low-risk areas in the western and north-eastern regions; the intermediate risk zones were located in the north-central region.
